# The Editor of the American Medical Gazette

**Published:** 1857-08

**Authors:** 


☞ The Editor of the American Medical Gazette, the able veteran of the American Medical Press, D. Meredith Reese, M. D., not connected with any Medical School, and presumed of course, to be an impartial observer. says, "The Atlanta Medical College has opened its Summer course of lectures with an increased class, and with so able, harmonious and enterprising a faculty, is sure of success.

"The opposition they have encountered from rival schools has only quickened their energies and hastened their prosperity. Prof. Means, and Professor Boring, made the speeches of the session at the late Nash ville Convention and secured many friends. Professor Westmoreland's correspondence with his Journal from Paris, is the best we have seen."

[The publication of the above is made without the knowledge of either of the gentlemen mentioned, and as we do not consider it as involving any individual laudation of ourselves, we have no hesitation in assuming the responsibility, considering it fully justified from that opposition to which allusion is made.—Sr. Ed.]

